# Optimization of Synthesis, Characterization and Cytotoxic Activity of Seleno-*Capparis spionosa* L. Polysaccharide

**DOI:** 10.3390/ijms131217275

**Published:** 2012-12-17

**Authors:** Yu-Bin Ji, Fang Dong, Lang Lang, Ling-Wen Zhang, Jing Miao, Zhen-Feng Liu, Li-Na Jin, Ying Hao

**Affiliations:** 1 Research Center on Life Sciences and Environmental Sciences, Harbin University of Commerce, Harbin 150076, China; E-Mails: dong1234fang@126.com (F.D.); lang8968@163.com (L.L.); lingwen2008@163.com (L.-W.Z.); 2 Engineering Research Center of Natural Anticancer Drugs, Ministry of Education, Harbin 150076, China; E-Mails: miaojing586@163.com (J.M.); huanwuxinde@163.com (Z.-F.L.); kinglena@126.com (L.-N.J.); xuefeng5233@yahoo.cn (Y.H.)

**Keywords:** seleno-*Capparis spionosa* L. polysaccharide(Se-CSPS), reaction optimization, response surface methodology, characterization, cytotoxic activity

## Abstract

In this study, an experiment was designed to optimize the synthesis of seleno-*Capparis spionosa* L. polysaccharide (Se-CSPS) by response surface methodology. Three independent variables (reaction time, reaction temperature and ratio of Na_2_SeO_3_ to CSPS) were tested. Furthermore, the thermal stability, particle size, shape and cytotoxic activity of Se-CSPS *in vitro* were investigated. The optimum reaction conditions were obtained shown as follows: reaction time 7.5 h, reaction temperature 71 °C, and ratio of Na_2_SeO_3_ to CSPS 0.9 g/g. Under these conditions, the Se content in Se-CSPS reached 5.547 mg/g, which was close to the predicted value (5.518 mg/g) by the model. The thermal stability, particle size and shape of Se-CSPS were significantly different from those of CSPS. Additionally, a MTT assay indicated that the Se-CSPS could inhibit the proliferation of human gastric cancer SGC-7901 cells in a dose-dependent manner.

## 1. Introduction

As an organic selenium compound, selenium polysaccharide (seleno-polysaccharide) maintains the basic configuration of the polysaccharide and physiological functions of selenium [1–5]. Moreover, seleno-polysaccharide can improve the bio-availability of selenium and reduce the toxicity and side effects of inorganic selenium [6]. Meanwhile, research shows that the biological activity of seleno-polysaccharide is higher than that of polysaccharide and selenium [7].

The natural seleno-polysaccharide is limited and scarce in plants, animals and micro-organisms [8]. It is necessary to increase the yield of seleno-polysaccharide by artificial methods. Biotransformation and chemical synthesis were feasible approaches for harvesting seleno-polysaccharide [9–12]. However, many defects of biotransformation can not be ignored, such as higher cost, longer time and lower yield. Therefore, chemical synthesis is the most effective way to get seleno-polysaccharide.

*Capparis spionosa* L., which is cultivated in China and central Asia, is a traditional medicinal plant [13]. Because *Capparis spionosa* L. contains various active ingredients (volatile oil, sugar ligands, glucose isothiocyanates and alkaloids), it is used for treating diseases such as rheumatoid arthritis, frozen shoulder and hypertension [14]. Our previous research has indicated that *Capparis spionosa* L. polysaccharide (CSPS) had anti-tumor activity *in vivo* and *in vitro*[15,16]. We found that CSPS could prolong the survival time of H22 bearing mice in a dose-dependent manner [14]. *In vitro*, under the laser scanning confocal microscope, we observed that the cytoplasm was leaked from intact HepG2 cells and the cells stained with AO/EB were disrupted to pieces in mid-CSPS and high-CSPS group [16]. Simultaneously, we discovered that CSPS could induce HepG2 cell apoptosis by increasing intracellular Ca^2+^[17].

Up to now, little information is available on the optimal conditions for seleno-*Capparis spionosa* L. polysaccharide (Se-CSPS) synthesis. In addition, there are no experiments to explore the thermal stability, particle size, shape and cytotoxic activity of Se-CSPS. Therefore, this paper employed a optimized by Box-Behnken design (BBD) and response surface methodology (RSM) [18–22] (three factors and three levels) to optimize the effects of reaction time, reaction temperature and ratio of Na_2_SeO_3_ to CSPS on the Se content in Se-CSPS. Furthermore, the thermal stability, particle size, shape and cytotoxic activity of Se-CSPS were evaluated for seeking high biological functional seleno-polysaccharide.

## 2. Results and Discussion

### 2.1. Single Factor Assays

**Figure 1:**
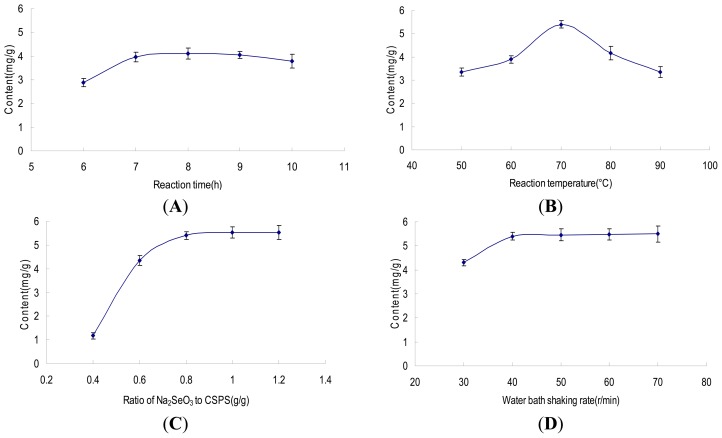
Effect of reaction time, temperature, ratio of Na_2_SeO_3_ to *Capparis spionosa* L. polysaccharide (CSPS) and water bath shaking rate on Se content. (**A**) reaction temperature 60 °C, ratio of Na_2_SeO_3_ to CSPS 0.8 g/g, water bath shaking rate 40 r/min; (**B**) reaction time 7 h, ratio of Na_2_SeO_3_ to CSPS 0.8 g/g, water bath shaking rate 40 r/min; (**C**) reaction time 7 h, reaction temperature 70 °C, water bath shaking rate 40 r/min; (**D**) reaction time 7 h, reaction temperature 70 °C, ratio of Na_2_SeO_3_ to CSPS 0.8 g/g.

The effects of single factors on Se content in Se-CSPS were shown in Figure 1. As seen from Figure 1A, the curve kept a mild slope after 7 h and the Se content decreased when the reaction time was 10 h. The reason for this result was that more and more monosaccharide and oligosaccharide were hydrolyzed from CSPS by acidic water for long time reaction. When the reacted solution was dialyzed with dialysis membrane in running water, the lower molecular weight monosaccharide and oligosaccharide were dialyzed out from solution. Se introduced into monosaccharide and oligosaccharide also was removed from dialysis membrane. This led to the decrease of Se content when the reaction time was 10 h. Therefore, 6–8 h was considered to be optimal reaction time in the designed experiment.

The Se content in Se-CSPS affected by different reaction temperature (50, 60, 70, 80, 90 °C) was shown in Figure 1B. Se content in Se-CSPS reached a maximum value at 70 °C. However, with the increase of reaction temperature after 70 °C, Se content was significantly decreased. The reason was similar to that of the reaction time. Because a certain percentage of CSPS was hydrolyzed to monosaccharide and oligosaccharide at the higher temperature, Se-monosaccharide and Se-oligosaccharide were dialyzed out from reacted solution through dialysis membrane. So, the 60–80 °C was used in the present work.

As seen from Figure 1C, the result indicated that Se content in Se-CSPS significantly increased at the ratio of Na_2_SeO_3_ to CSPS 0.8 g/g and then maintained a dynamic equilibrium. Therefore, the ratio of Na_2_SeO_3_ to CSPS ranges of 0.6–1.0 g/g were adopted for the reaction. Figure 1D showed that Se content in Se-CSPS obvious increased with the increase of water bath shaking rate, but there was no significant increase after 40 r/min. This meant that 40 r/min was sufficient for the reaction. Herein, 40 r/min was determined as the reaction water bath shaking rate in next optimization experiments.

### 2.2. Optimization by RSM

#### 2.2.1. Results of Selenylation of Optimization of the Procedure

Seventeen experimental conditions for optimizing the three individual parameters were designed in BBD (Table 1). Five replicates (Exp. No. 13–17) at the center of the design were used to allow for estimation of a pure error sum of squares. The response value in each trial was the average of triplicates.

The Se content in Se-CSPS showed consideration variation with the reaction conditions (Table 1). As Table 1 shown, Se content in Se-CSPS ranged from 2.438 to 5.545 mg/g.

#### 2.2.2. Model Fitting and Statistical Significance Analysis

By applying multiple regression analysis on the experimental data, the relationship between response variables and the test variables was obtained from the following second-order polynomial equation.

$$Y=5.36+0.26X_{1}+0.43X_{2}+0.28X_{3}-0.46X_{1}X_{2}+0.27X_{1}X_{3}+0.33X_{2}X_{3}-0.30{X_{1}}^{2}-1.30{X_{2}}^{2}-0.56{X_{3}}^{2}$$

*p*-Value is a tool to check the significance of each coefficient, which in turn may indicate the pattern of the interactions among the variables. Table 2 showed that Se content in Se-CSPS was significantly affected by three variables (*p* < 0.05). It was evident that the quadratic parameters (*X*_1_^2^, *X*_2_^2^, *X*_3_^2^) and the interactive parameters(*X*_1_*X*_2_, *X*_2_*X*_3_) were significant at the 0.05 level, whereas the interactive parameter (*X*_1_*X*_3_) was not significant (*p* > 0.05). Meanwhile, the determination coefficient (*R*^2^ = 0.9689) of the model indicated that only 3.11% of the total variations could not be explained by the calculated model. A low value 0.0577 of coefficient of the variation (C.V.) implied a high degree of precision and a good deal of reliability of the experimental values. This meant that the model could be used to analyze and predict selenylation process results.

As Table 3 shown, *F*-value and *p*-value (*F* = 24.21, *p* = 0.0002) of the model, with no significant lack of fit at *p* > 0.05, indicated that the model used to fit response variables was extremely significant and adequate to represent the relationship between the response and the independent variables.

#### 2.2.3. Optimization of Selenylation Conditions

Response surface methodology plays a key role in identifying the optimum values of the independent variables efficiently, under which dependent variables could achieve a maximum response.

**Figure 2:**
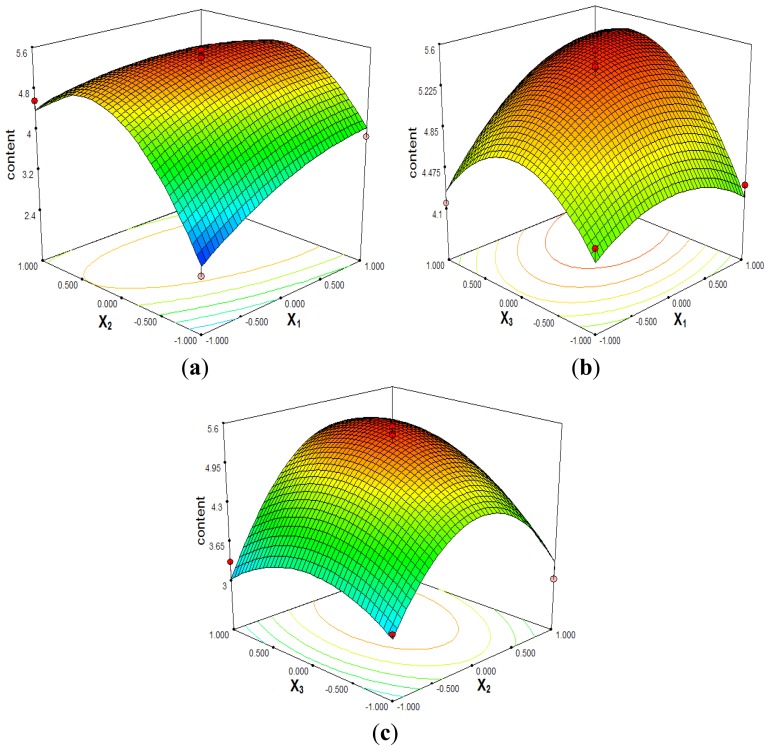
Response surface plots (3D) of reaction time, reaction temperature and ratio of Na_2_SeO_3_ to CSPS (*X*_1_: reaction time; *X*_2_: reaction temperature; *X*_3_: ratio of Na_2_SeO_3_ to CSPS).

Based on the regression equation obtained by Design-Expert 7.0 (State-Ease, Inc., Minneapolis, MN, USA), Se contents in Se-CSPS affected by *X*_1_, *X*_2_ and *X*_3_ were graphically presented by 3D response surface in Figure 2.

The response surface analysis predicted that the optimal reaction time, reaction temperature and ratio of Na_2_SeO_3_ to CSPS were 7.53 h, 71.26 °C and 0.88 g/g, respectively. Meanwhile, the model predicted the optimized Se content in Se-CSPS was 5.518 mg/g.

The interaction between reaction time (*X*_1_) and reaction temperature (*X*_2_) on the Se content in Se-CSPS was shown in Figure 2a (*p* = 0.0083 < 0.05, Table 2). At a definite ratio of Na_2_SeO_3_ to CSPS, the Se content significantly enhanced with reaction time ranging from 6 h to 8 h and reaction temperature from 50 °C to 70 °C. Likewise, when the reaction time ranged from 6 h to 8 h and the ratio of Na_2_SeO_3_ to CSPS varied between 0.6 g/g and 1.0 g/g, the Se content in Se-CSPS was increasing gradually (Figure 2b). However, the interactive effect between reaction time and the ratio of Na_2_SeO_3_ to CSPS was not significant (*p* = 0.0685 > 0.05, Table 2). As shown in Figure 2c and Table 2, the interaction of reaction temperature (*X*_2_) and ratio of Na_2_SeO_3_ to CSPS (*X*_3_) had a much higher effect on the selenylation (*p* = 0.0335 < 0.05).

#### 2.2.4. Verification of Predictive Model

In order to validate the accuracy and reliability of the model equation for predicting the optimum response value, the validation experiments were performed in triplicate. Taking fully into account the actual operating convenience, the confirmatory experimental verification was tested under the following conditions: reaction time 7.5 h, reaction temperature 71 °C and ratio of Na_2_SeO_3_ to CSPS 0.9 g/g. A mean value of (5.547 ± 0.09) mg/g (*n* = 3) was obtained from the confirmatory experiment. The results showed that the actual value was very close to the predicted result. It also indicated the actual optimization parameters were reliable (Table 4).

### 2.3. Characterization of Se-CSPS

#### 2.3.1. Thermal Weight Loss Analysis

**Figure 3:**
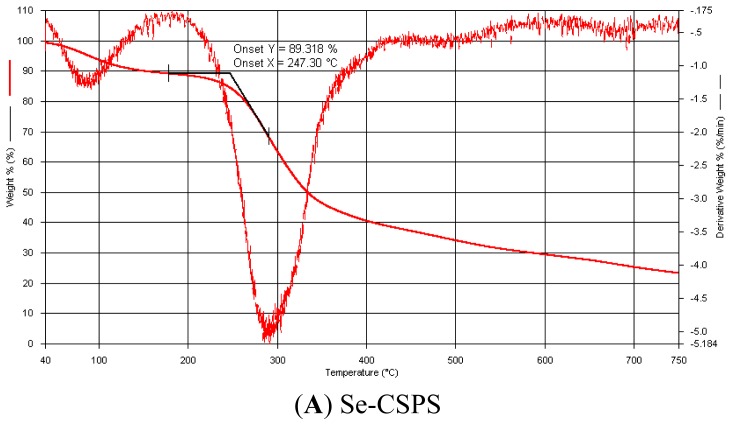

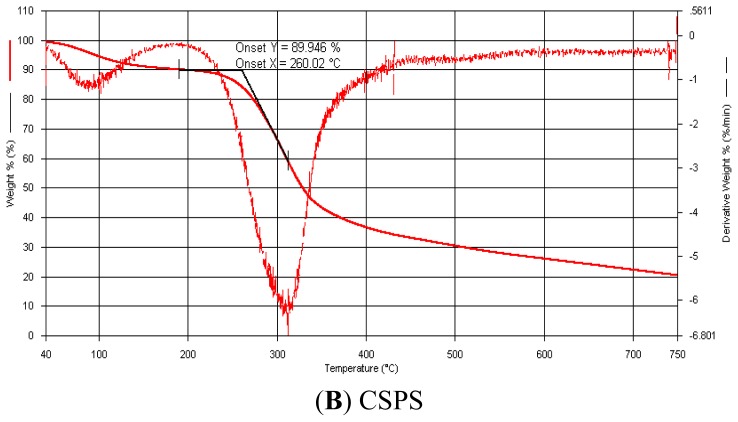
TG and DTG curves recorded for (**A**) Se-CSPS and (**B**) CSPS.

The results of TG and DTG of Se-CSPS and CSPS were illustrated in Figure 3. TG and DTG spectrum shapes of Se-CSPS and CSPS were similar. The first peaks of them were dehydration peak. In the second peak, both of them rapidly lost weight amounting to 50% in weight loss process. This indicated that Se-CSPS and CSPS took place violent decomposition reaction in the temperature range.

However, minor differences between Se-CSPS and Se could be seen from the curves. On the one hand, the temperature of dehydration peak (5% loss, around 85 °C) of Se-CSPS was significant lower than that of CSPS (5% loss, around 92 °C). On the other hand, in the decomposition peak (200–400 °C), the fastest weight loss temperatures of Se-CSPS and CSPS were 283.95 °C and 311.86 °C, respectively. This demonstrated that stability of Se-CSPS was lower than that of CSPS.

**Figure 4:**
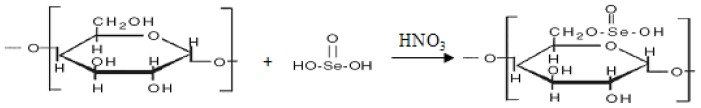
Selenylation mechanism of seleno-polysaccharide.

This phenomenon may be explained by chemical bonds and molecular orbital theory [23]. The studies conformed that the 6′-OH in polysaccharide could be substituted by HSeO^3 −^ in selenylation (Figure 4) [10,24].

When HSeO_3_^−^ was introduced into CSPS and substituted 6′-OH, p orbital of oxygen and Se=O bond formed the p–π conjugated system in which the electron cloud of C–O transferred to Se=O. This caused that C–O bond energy in Se-CSPS was lower than that of CSPS and C–O bond in Se-CSPS could be broken more easily. Therefore, the lower C–O bond energy in Se-CSPS caused that HSeO_3_^−^ group was easily removed from Se-CSPS in the lower temperature. Further, the removal of HSeO_3_^−^ accelerated the Se-CSPS decomposition.

#### 2.3.2. Particle Size Distribution

**Figure 5:**
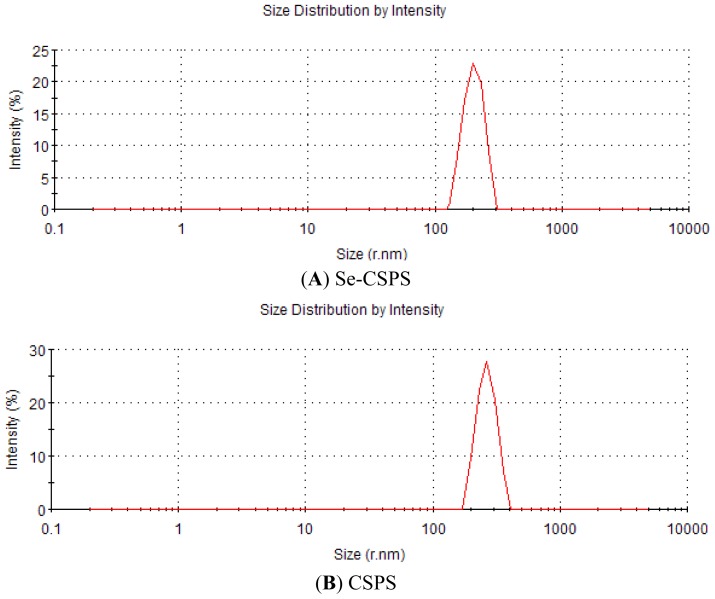
Particle size distribution curves recorded for (**A**) Se-CSPS and (**B**) CSPS.

The pharmacological activity of polysaccharide depended on the particle size [25]. Generally, particle with the smaller size could be easily absorbed and utilized by body. As seen from Figure 5, the average particle size of Se-CSPS (203 nm) was significant smaller than that of CSPS (266 nm) at the same concentration. The reason was that CSPS was hydrolyzed into some smaller pieces by acidic water in selenylation. It implied that absorption and utilization of Se-CSPS were better than that of CSPS *in vivo* and *in vitro* at the same dose.

#### 2.3.3. Shape in SEM

**Figure 6:**
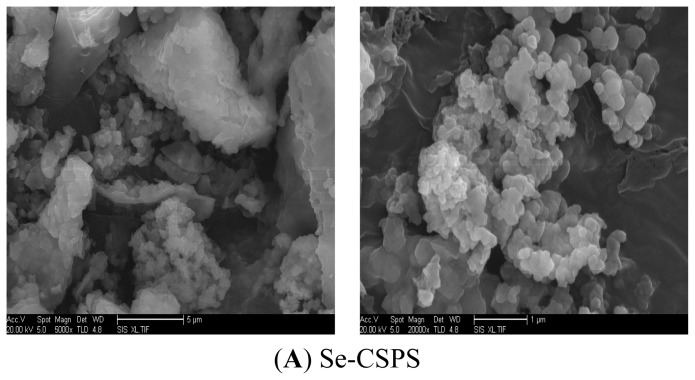

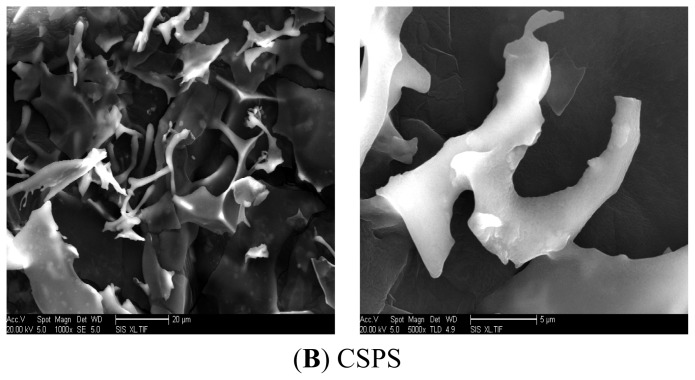
Scanning electron micrographs of Se-CSPS and CSPS.

Scanning electron microscopy (SEM) has been used to identify the sample microscopic appearance. As shown in Figure 6, the three-dimensional image of Se-CSPS was different from CSPS. It could be seen from Figure 6A that the shape of Se-CSPS was cylindrical or spherical. Compared with Se-CSPS, the shape of CSPS presented long strip and rod. The changes of shape indicated the chemical change in most cases. This suggested that the chemical structure might change after selenylation.

### 2.4. MTT Assay

**Figure 7:**
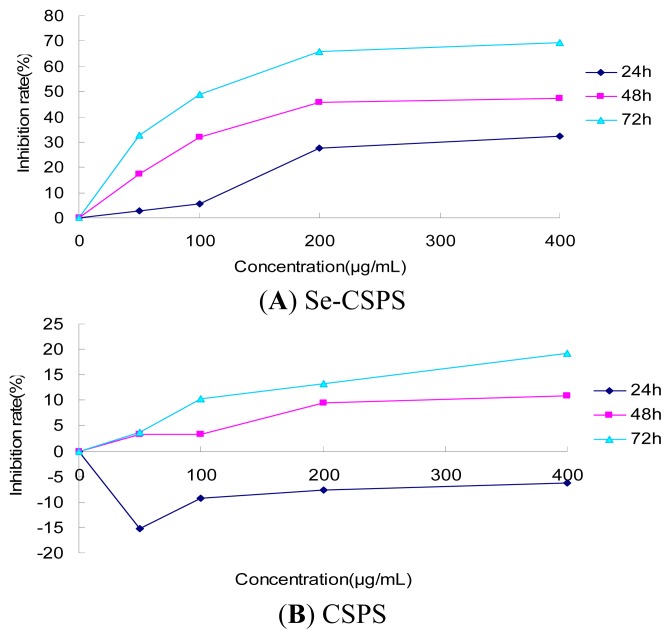

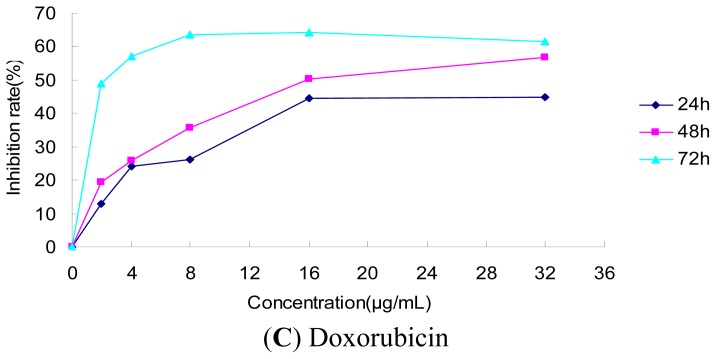
Inhibition effect of (**A**) Se-CSPS; (**B**) CSPS and (**C**) doxorubicin on SGC-7901.

As Figure 7A shown, the inhibition rate of Se-CSPS significantly raised with the increase of dose. In addition, the inhibition rate of Se-CSPS also remarkably increased with the time prolongation. For example, the maximum inhibition rate of Se-CSPS at 24, 48, 72 h attained to 32.12%, 47.22% and 69.49%, respectively. The inhibition rate of Se-CSPS was in dose-dependent and time-dependent manner, and was higher than that of CSPS (Figure 7B). Additionally, CSPS with the low dose could temporarily induce cell proliferation at 24 h. The inhibition rate of doxorubicin (positive group) was shown in Figure 7C. Simultaneously, IC_50_ of Se-CSPS (111.90 μg/mL) was obtained from the inhibition rate. The result indicated that the Se-CSPS could inhibit the proliferation of human gastric cancer SGC-7901 cells. This also suggested that seleno-polysaccharide obtained from selenylation could significantly increased cytotoxicity to tumor cells.

## 3. Experimental Section

### 3.1. Materials and Instrument

The fruit of *Capparis spionosa* L. was purchased from Xinjiang Wanbang Biotechnology Co., Ltd., China. Dehydrated alcohol, acetone, ligarine, hydrogen peroxide, nitric acid, ascorbic acid, 1, 2-diaminobenzene, toluene (analytical grade) were purchased from Tianjin FuChen Chemical Reagent Factory, China. Activated carbon was from Boyuan Activated Carbon Factory, China. Papain (>500,000 U/g) was provided by Obo Star Biotechnology Co., Ltd., China. Na_2_SeO_3_ was obtained from Tianjin BASF Chemical Trading Co., Ltd., China. Na_2_CO_3_ was purchased from Shanghai ShanPu Chemical Co., Ltd., China. Dialysis membrane was supplied by Beijing Solarbio Science & Technology Co., Ltd., China.

SP-752 UV-Vis Spectrophotometer was purchased from Shanghai Spectrum Instruments Co., Ltd., China. Pyris 6-thermal gravimetric analysis instrument was obtained from Perkin Elmer Co., Ltd., USA. Zetasizer Nano ZS90 nano-particle size and zeta potential analyzer was purchased from British Malvern Instruments Co., Ltd., UK. QUANTA 200-scanning electron microscope was purchased from FFI Co., Ltd., USA. WELLSCAN MK 3 Microplate Reader was obtained from Bio-Rad Co., Ltd., USA.

### 3.2. Extraction, Purification and Isolation of CSPS

The dried fruit of *Capparis spionosa* L. (2500 g) was ground in a blender to obtain a fine powder. The powder was extracted with 95% ethanol (5000 mL, ×3 cycles) at 90 °C for 3 h, and filtered through nylon cloth (pore diameter 38 μm). The residue was dried under reduced pressure and extracted by water bath in extraction temperature 90 °C, extraction time 120 min, ratio of water to sample 25 mL/g, extraction cycles 3. The extraction solution was separated from insoluble residue by centrifuge (4000× *g* for 5 min, at 20 °C), and then precipitated by dehydrated alcohol to a final concentration of 80% (*v*/*v*). The crude polysaccharide was collected by centrifuge (4000× *g* for 10 min, at 20 °C). Protein of crude polysaccharide was discarded by 0.1% (*g*/*v*) papain and Sevag. Pigment was treated with activated carbon and H_2_O_2_. CSPS collected by precipitation with dehydrated alcohol to the concentrations 80% (*v*/*v*) was washed with dehydrated alcohol, acetone and ligarine, vacuum dried.

### 3.3. Selenylation

Different ratios of Na_2_SeO_3_ and CSPS were dissolved by HNO_3_ (100 mL, 0.05%) in erlenmeyer flasks. Then, the mixed solution were reacted in the designed reaction time, reaction temperature and water bath shaking rate. After the reaction, Na_2_CO_3_ was added into the reacted solution to adjust pH to 5–6. The solution was centrifuged to remove the insoluble residue (3000× *g* for 5 min, at 20 °C), and then dialyzed with dialysis membrane in running water. Ascorbic acid was used to detect whether free SeO_3_^2−^ was dialyzed out from the solution. When ascorbic acid did not redden the solution, the Se-CSPS solution was collected, freeze-dried.

### 3.4. Detection of Se Content in Se-CSPS

Adjusted pH to 1.5–2.5 by 80% formic acid, 1 mL drawn from different concentration Se standard solution (0.5, 1.5, 2.5, 3.5, 4.5, 5.5, 5.5, 7.5 μg/mL) was added into 5 mL 1, 2-diaminobenzene (0.1%), respectively. The mixed solution was added up to 25 mL by pure water (reaction in dark, 50 min). Then, 10 mL toluene was added into 25 mL solution to extract Se. Finally, toluene solution was detected by UV-Vis spectrophotometer under 334 nm. The calibration curve was shown as follows: *Y* = 0.0197*X* + 0.0343, *R*^2^ = 0.9998.

100.00 mg Se-CSPS was hydrolyzed by 2.00 mL mixed acid (HNO_3_:H_2_SO_4_:HClO_4_ = 4:1:1) for 24 h. Then, detection was followed the above method. The Se content in Se-CSPS was calculated according to the calibration curve.

### 3.5. Design of Optimization

According to the results of mono-factor experiments for the Se-CSPS production, the proper ranges of reaction temperature, reaction time, ratio of Na_2_SeO_3_ to polysaccharide were determined preliminarily. Three independent variables in a BBD (*X*_1_, reaction time; *X*_2_, reaction temperature; *X*_3_, ratio of Na_2_SeO_3_ to CSPS) at three levels were performed. Table 5 showed that the range of independent variables and their levels.

### 3.6. Characterization Detection of Se-CSPS

#### 3.6.1. Determination of Thermal Weight Loss Analysis

Se-CSPS and CSPS were detected by Pyris 6-thermal gravimetric analysis instrument [24]. (Air flow rate: 5 mL/min, heating rate: 10 °C/min, scope of temperature: 20–760 °C).

#### 3.6.2. Detection of Particle Size Distribution

The solution of Se-CSPS and CSPS in water (0.02 mg/mL) was detected by Zetasizer Nano ZS90 nano-particle size and zeta potential analyzer. All data was analyzed by software of particle size distribution.

#### 3.6.3. Observation under Scanning Electron Microscopy

The powder of Se-CSPS and CSPS coated by Au was fixed on the conductive adhesive. The three-dimensional images in different resolutions were observed by QUANTA 200-scanning electron microscope.

### 3.7. MTT Detection of Se-CSPS *in vitro*

Human gastric cancer SGC-7901 cells in logarithmic growth phase were digested by 0.25% trypsin for the preparation of cell suspension (1 × 10^4^ cells/mL). Cells were cultured in 96 well plates (1 × 10^3^ cells/well) overnight prior to the treatment. Then, the cells were treated with Se-CSPS, CSPS (50, 100, 200, 400 μg/mL), doxorubicin (2, 4, 8, 16, 32 μg/mL, positive group) and RPMI-1640 medium (control group), 100 μL every well, six parallel holes every group. After 24, 48, 72 h, supernatant of the holes was discarded and 100 μL MTT of 0.5 mg/mL in serum-free medium was added in the hole. Cultured for 4 h, 200 μL DMSO was added into per hole after discarding supernatant. OD value of each hole under detection wavelength 570 nm, reference wavelength of 490 nm was detected by MK3 type microplate reader. The ratio of inhibition of Se-CSPS was calculated according to the following formula and the IC_50_ were obtained from the ratio of inhibition.

Ratio of inhibition (%)=(1-OD value of Se-CSPS group/OD value of control group)×100

### 3.8. Statistical Analysis

Design-Expert (Version 7.0, State-Ease, Inc., Minneapolis, MN, USA) software was used to analyze the experimental data. Statistical comparison within groups was carried out by one way ANOVA. A *p*-value of less than 0.05 was considered to be significant statistically. All determinations were carried out in triplicate.

## 4. Conclusions

In this paper, the selenylation conditions for Se-CSPS were optimized by BBD, and a quadratic polynomial model was obtained from RMS. The confirmatory experimental optimum conditions of Se-CSPS were as follows: reaction time 7.5 h, reaction temperature 71 °C and the ratio of Na_2_SeO_3_ to CSPS 0.9 g/g. The optimal Se content of (5.547 ± 0.09) mg/g obtained from confirmatory experiments closely matched the predicted Se content of 5.518 mg/g. Furthermore, the thermal stability, particle size and shape of Se-CSPS were significantly different from those of CSPS. In addition, Se-CSPS obtained from selenylation could significantly increased cytotoxic activity. Further research on the chemical structure and anti-tumor mechanism of Se-CSPS will be carried out in the future.

## Figures and Tables

**Table 1:** Box-Behnken design matrix of three variables and the experimental observed responses.

Exp. No.	*X*_1_/Reaction time (h)	*X*_2_/Reaction temperature (°C)	*X*_3_/Ratio of Na_2_SeO_3_ to CSPS (g/g)	Se content in Se-CSPS (mg/g)
1	−1 (6)	−1 (60)	0 (0.8)	2.438
2	1 (8)	−1 (60)	0 (0.8)	3.874
3	−1 (6)	1 (80)	0 (0.8)	4.574
4	1 (8)	1 (80)	0 (0.8)	4.183
5	−1 (6)	0 (70)	−1 (0.6)	4.348
6	1 (8)	0 (70)	−1 (0.6)	4.319
7	−1 (6)	0 (70)	1 (1.0)	4.149
8	1 (8)	0 (70)	1 (1.0)	5.199
9	0 (7)	−1 (60)	−1 (0.6)	3.186
10	0 (7)	1 (80)	−1 (0.6)	3.022
11	0 (7)	−1 (60)	1 (1.0)	3.317
12	0 (7)	1 (80)	1 (1.0)	4.476
13	0(7)	0 (70)	0 (0.8)	5.545
14	0 (7)	0 (70)	0 (0.8)	5.423
15	0 (7)	0 (70)	0 (0.8)	5.399
16	0 (7)	0 (70)	0 (0.8)	5.220
17	0 (7)	0 (70)	0 (0.8)	5.234

**Table 2:** Test result of significance for regression coefficients.

Parameter	Estimate	Sum of squares	Standard error	df	*F*-value	*p*-value
intercept	5.36		0.11	1		
*X*_1_	0.26	0.53	0.089	1	8.49	0.0226
*X*_2_	0.43	1.48	0.089	1	23.53	0.0019
*X*_3_	0.28	0.64	0.089	1	10.21	0.0152
*X*_1_*X*_2_	−0.46	0.83	0.13	1	13.27	0.0083
*X*_1_*X*_3_	0.27	0.29	0.13	1	4.63	0.0685
*X*_2_*X*_3_	0.33	0.44	0.13	1	6.96	0.0335
*X*_1_^2^	−0.30	0.37	0.12	1	5.90	0.0455
*X*_2_^2^	−1.30	7.12	0.12	1	113.21	<0.0001
*X*_3_^2^	−0.56	1.34	0.12	1	21.28	0.0024

*R*^2^ = 0.9689; Adjusted *R*^2^ = 0.9289; C.V. = 0.0577.

**Table 3:** Analysis of variance for fitted quadratic polynomial model.

Source	Sum of squares	df	Mean square	*F*-value	*p*-value	
Model	13.70	9	1.52	24.21	0.0002	significant
Residual	0.44	7	0.063			
Lack of fit	0.37	3	0.12	6.48	0.0514	not significant
Pure error	0.075	4	0.019			
Cor total	14.14	16				

**Table 4:** Predicted and experimental values of the responses under optimum conditions.

	Reaction time (h)	Reaction temperature (°C)	Ratio of Na_2_SeO_3_ to CSPS (g/g)	Se content in Se-CSPS (mg/g)
Predicted conditions	7.53	71.26	0.88	5.518
Experimental conditions	7.5	71	0.9	5.547 ± 0.09 *

* Mean ± SD (n = 3).

**Table 5:** Independent variables and their levels used in the response surface design.

Independent variables	Factor level		
	−1	0	1
X_1_ reaction time (h)	6	7	8
X_2_ reaction temperature (°C)	60	70	80
X_3_ ratio of Na_2_SeO_3_ to CSPS (g/g)	0.6	0.8	1
